# Synthetic T1 mapping using artificial intelligence and oxygenation-sensitive cardiovascular magnetic resonance for myocardial tissue characterization in hypertrophic cardiomyopathy and cardiac amyloidosis

**DOI:** 10.3389/fcvm.2026.1855952

**Published:** 2026-07-28

**Authors:** Faezeh Lotfikazemi, Mitchel Benovoy, Michael Chetrit, Judy Luu, Leila Haririsanati, Moezedin Javad Rafiee, Matthias G. Friedrich

**Affiliations:** 1 Department of Experimental Medicine, McGill University, Montreal, QC, Canada; 2 Area19 AI Inc., Montreal, QC, Canada; 3 Division of Cardiology, McGill University, Montreal, QC, Canada; 4 Research Institute of the McGill University Health Centre (RI-MUHC), Montreal, QC, Canada

**Keywords:** cardiac amyloidosis, hypertrophic cardiomyopathy, oxygenation-sensitive cardiovascular magnetic resonance, synthetic T1 mapping, generative adversarial network, artificial intelligence, myocardial tissue characterization, cardiac MRI

## Abstract

**Background:**

Cardiovascular magnetic resonance (CMR) techniques provide detailed myocardial tissue characterization. However, LGE requires the administration of contrast agents, while T1/T2 mapping involves prolonged acquisition times, sensitivity to motion artifacts, and protocol complexity. LGE can also be limited in differentiating cardiac amyloidosis from hypertrophic cardiomyopathy (HCM) due to overlapping enhancement patterns. Oxygenation-sensitive CMR (OS-CMR), offers a rapid, contrast-free alternative but lacks direct quantitative outputs.

**Methods:**

We propose a unified AI framework integrating DeepOxyMap and a Residual Generative Adversarial Network (R-GAN) to extract latent features and generate synthetic T1 maps from OS-CMR. DeepOxyMap was trained on 117 OS-CMR images using a VGG19-based architecture to identify fibrosis-related myocardial patterns via heatmaps. The R-GAN was subsequently trained on 2,044 OS-CMR images from two independent cohorts to generate T1-parametric maps directly from OS-CMR. Performance was assessed using image similarity metrics, ROI-based clinical validation, T1 recovery curve analysis, and Extended Phase Graph simulations.

**Results:**

DeepOxyMap achieved an accuracy of of 83.5%, with a precision of 85.5% and a recall of 79.7%.AUC values were 0.93 (ICMP), 0.88 (*N*ICMP), and 0.97 (healthy). Heatmaps showed strong spatial correspondence with fibrosis regions on LGE images. In the second stage, the R-GAN outperformed Pix2Pix (SSIM: 0.810, PSNR: 16.01, PCC: 0.748). EPG-based validation demonstrated near-perfect agreement between synthetic and theoretical T1 recovery curves (*R*^2^ > 0.9994). T1 curve agreement was also strong in ROI-based analysis. HCM: MAE = 0.0970, MSE = 0.0106, RMSE = 0.1030, *R*^2^ = 0.7764, Pearson = 0.9658, Spearman = 0.9286. Amyloidosis: MAE = 0.0988, MSE = 0.0141, RMSE = 0.1187, *R*^2^ = 0.8599, Pearson = 0.9718, Spearman = 0.9762. Synthetic T1 maps preserved disease-specific signal patterns. Synthetic and native T1 values demonstrated excellent agreement (*r* = 0.976, *p* < 0.001) with minimal bias (−6.7 ms).

**Conclusion:**

This study demonstrates that OS-CMR contains latent tissue information that can be leveraged to generate quantitative T1 maps using AI. The proposed contrast-free framework enables rapid acquisition, eliminates the need for gadolinium, and preserves clinically relevant myocardial patterns, offering a promising approach for non-invasive cardiac tissue characterization in clinical practice.

## 1 Introduction

Cardiovascular magnetic resonance (CMR) has become a cornerstone in detecting and characterizing myocardial disease (1, 2), largely due to its ability to directly visualize myocardial fibrosis (8). The presence and pattern of fibrosis are critical in a wide spectrum of cardiomyopathies, from ischemic scars after myocardial infarction to non-ischemic fibrotic changes in cardiomyopathies, and infiltrative deposits in diseases like cardiac amyloidosis (9). Late Gadolinium Enhancement (LGE) CMR, which uses a gadolinium contrast agent to highlight scar tissue (3), is widely regarded as the reference standard for non-invasive imaging of myocardial scar and focal fibrosis (4). By revealing hyperintense (bright) areas of retained contrast in injured myocardium against nulled normal myocardium, LGE enables clinicians to identify even small scars with high spatial resolution. Importantly, LGE findings aid in differentiating ischemic versus non-ischemic cardiomyopathy based on scar location and morphology: ischemic injuries classically appear subendocardial or transmural in coronary artery territories (10), whereas non-ischemic patterns (mid-wall stripes in dilated cardiomyopathy, patchy fibrosis in myocarditis or sarcoidosis, subepicardial or septal scars in hypertrophic cardiomyopathy, etc.) (11). These fibrosis patterns on CMR often have diagnostic and prognostic significance. This, CMR's unique capability to depict fibrosis, has made it indispensable in the workup of cardiomyopathies.

Beyond focal scar detection, CMR also allows identification of diffuse fibrotic or infiltrative processes that may not be evident as discrete LGE lesions. Native T1 mapping has emerged as a powerful complementary technique in this regard. Unlike LGE, which provides a binary contrast-enhanced image of scar vs. normal tissue, T1 mapping quantitatively measures the T1 relaxation time in each voxel of myocardium without requiring wcontrast (12). Elevated native T1 values signify alterations in tissue composition such as fibrosis, edema, or amyloid deposition. Native T1 mapping and its derivative extracellular volume (ECV) mapping (13) have shown particular utility in diseases with diffuse myocardial involvement that LGE might miss. For instance, in cardiac amyloidosis, often considered a paradigm of diffuse interstitial infiltration LGE CMR can be challenging to interpret because the entire myocardium may enhance nearly uniformly, making it hard to distinguish normal from abnormal myocardium (4). The diffuse subendocardial gadolinium uptake in amyloid leads to difficulty “nulling” the myocardium on LGE images, often resulting in confusing or false-negative scans (14). In contrast, T1 mapping circumvents this limitation by providing an objective pixel-wise measure of tissue characteristics without reliance on nulling, and it robustly detects the widespread interstitial expansion in amyloidosis (4, 14). Both AL (light-chain) and ATTR (transthyretin) amyloid cardiomyopathy exhibit markedly elevated native T1 values, to the extent that T1 mapping can reliably distinguish cardiac amyloidosis from other causes of increased wall thickness such as hypertrophic cardiomyopathy (14, 15). More broadly, native T1 mapping has demonstrated promise as a novel biomarker for myocardial tissue characterization: it can identify diffuse fibrosis that eludes LGE, support diagnosis and risk stratification in both ischemic and non-ischemic heart disease, and even track disease progression or regression over time with serial measurements. Therefore, LGE and T1 mapping together represent the state-of-the-art CMR techniques for fibrosis imaging, LGE being the clinical gold standard for focal scar identification, and T1 mapping emerging as an important tool for diffuse fibrosis and infiltrative cardiomyopathies. These methods have significantly improved our ability to detect myocardial fibrosis across various cardiomyopathies, which is crucial since fibrosis burden often correlates with symptoms, ventricular dysfunction, and outcomes in heart disease.

While LGE is invaluable, LGE is invaluable for clinical decision-making, it comes with notable limitations that motivate the search for needle-free, contrast-free imaging alternatives (1). A central concern is the use of gadolinium-based contrast agents (GBCAs). In patients with advanced renal dysfunction, GBCAs are contraindicated due to the risk of nephrogenic systemic fibrosis (NSF), a rare but serious systemic disorder triggered by gadolinium retention in tissues (16). Even in patients with normal kidneys, recent findings have shown that gadolinium can accumulate in the brain and other organs after repeated MRI exams, raising questions about long-term safety (16). Eliminating the need for contrast not only averts these risks but also simplifies workflow (no IV line or injection needed) and reduces cost and scan time. Another limitation of LGE is that it primarily detects focal fibrosis, where normal myocardium abuts scar tissue; it may fail to reveal diffuse fibrosis because there is no normal reference myocardium to create the contrast differential (13). In diseases with diffuse myocardial involvement (e.g., diffuse interstitial fibrosis from long-standing hypertension or infiltrative disease in early amyloid), an LGE image can appear deceptively “normal” despite significant microscopic fibrosis, since the signal of diseased tissue blends with the equally abnormal “reference” tissue (17). T1 mapping addresses part of this shortcoming by quantifying tissue changes without requiring a normal reference region, but standard T1 mapping still necessitates specialized sequences and, for ECV calculation, additional contrast-enhanced scans (12). Hence, there is a strong impetus to develop entirely non-contrast CMR methods that can replicate the diagnostic value of LGE for fibrosis detection by developing advanced AI models like VNE (18). Such methods would be needle-free (no IV cannula or contrast injection), safer for vulnerable patients, and amenable to repeated use.

Oxygenation-sensitive CMR (OS-CMR) is a contrast-free imaging technique (5) based on modified steady-state free-precession (mSSFP) sequences acquired during breathing maneuvers (19). It captures myocardial oxygenation dynamics and can be acquired in approximately one minute. Despite these advantages, OS-CMR lacks direct quantitative outputs, limiting its application for myocardial tissue characterization.

One promising approach to achieving contrast-free fibrosis imaging lies in the application of advanced artificial intelligence, like generative adversarial networks (GANs) (20). GANs, first introduced by Goodfellow et al. in 2014 (21), are a class of deep learning models including generator and discriminator competition, capable of learning complex data distributions and producing realistic synthetic images (14). In a GAN framework, a generator network is trained to create fake images (for example, an MRI sequence) from input data, while a discriminator network learns to distinguish the generator's fakes from real images like a simple classifier; through their adversarial training dynamic, the generator progressively improves until its synthetic outputs are indistinguishable from real images to the discriminator. This powerful approach has been rapidly adopted in medical imaging research for tasks involving image-to-image translation (22) and modality transformation (23) and even to generate missing or enhanced contrasts within MRI (24). In the context of CMR, generative models offer the potential to predict contrast-enhanced representations from non-contrast data. For example, the Virtual Native Enhancement (VNE) approach, developed at the University of Oxford, leverages deep learning to synthesize LGE-like images by integrating information from native cine and T1 mapping sequences. This method demonstrates that contrast-equivalent imaging can be inferred from non-contrast acquisitions of the same heart (18, 25). The results have been encouraging in patients with prior myocardial infarction; the VNE-generated images showed scar in the same locations as real LGE images (18). These findings demonstrate that GANs can successfully capture the complex features of fibrosis on non-contrast images and output a clinically useful approximation of a contrast-enhanced scan. These advances in deep learning models highlight a new window in CMR: using deep generative models to synthesize diagnostic images or maps that traditionally required invasive contrast injections (6, 7).

Motivated by the need for contrast-free myocardial tissue characterization, this study aims to develop a technique capable of providing fibrosis-related information comparable to LGE without the use of gadolinium-based contrast agents. In our prior work, DeepOxyMap, recently published in the *Journal of Cardiovascular Magnetic Resonance* (26), demonstrated that oxygenation-sensitive CMR (OS-CMR) contains latent myocardial features associated with fibrosis and cardiomyopathy classification. These findings suggest that contrast-free cine imaging may encode diagnostically relevant tissue characteristics traditionally assessed using LGE or T1 mapping. Building on this foundation, we propose a unified artificial intelligence framework that integrates DeepOxyMap—specifically adapted to focus on myocardial patterns in hypertrophic cardiomyopathy (HCM) and cardiac amyloidosis, which represent particularly challenging cases for feature extraction and interpretation—with a novel Residual Generative Adversarial Network (R-GAN) for the synthesis of quantitative T1 parametric maps. Unlike prior approaches that rely on multiple imaging sequences, the proposed framework operates using a single oxygenation-sensitive CMR (OS-CMR) modality and focuses on reconstructing disease-specific myocardial patterns. We hypothesize that OS-CMR inherently encodes sufficient latent information to enable accurate reconstruction of quantitative myocardial tissue properties, thereby providing a fully contrast-free pathway for myocardial tissue characterization.

## 2 Material and method

### 2.1 Data acquisition

To support the two-stage framework, separate datasets were used for DeepOxyMap and the R-GAN synthesis pipeline. For DeepOxyMap, a clinically curated subset of 131 subjects was selected from the available cohorts to investigate whether oxygenation-sensitive CMR (OS-CMR) contains disease-specific myocardial features. Following quality control, 14 subjects were excluded because of image artifacts, incomplete clinical information, or insufficient image quality, resulting in a final study cohort of 117 subjects consisting of 68 healthy controls, 25 ischemic cardiomyopathy (ICMP) cases, and 24 non-ischemic cardiomyopathy (NICMP) cases. The NICMP category included hypertrophic cardiomyopathy (HCM), cardiac amyloidosis. These data were used exclusively for DeepOxyMap training, validation, testing, and feature interpretation.

For quantitative T1 map synthesis, a substantially larger cohort was assembled from the Courtois Cardiovascular Signature (CV-SIG) Program and the CMR-10 study. Initially, 2,049 subjects with matched OS-CMR and native T1 acquisitions were identified. Twenty-three subjects were excluded during preprocessing because of severe imaging artifacts, missing sequences, or slice-matching inconsistencies, yielding a final cohort of 2,026 subjects (4,052 paired OS-CMR and T1-weighted images). The CV-SIG cohort was primarily used for model development and training (*n* = 1,635), while an independent fibrosis cohort from the CMR-10 study (*n* = 155) was reserved for validation and testing. This separation ensured that model performance was evaluated on unseen data acquired independently from the training cohort (Table 1). The CV-SIG cohort encompassed a broad spectrum of myocardial tissue characteristics, including subjects with elevated native T1 values associated with fibrosis, infiltration, and non-ischemic myocardial abnormalities. In addition to quantitative validation, independent disease-specific validation was performed using 20 hypertrophic cardiomyopathy (HCM) and cardiac amyloidosis cases. These conditions were selected because they are characterized by distinct patterns of myocardial remodeling and elevated native T1 values, providing a clinically relevant assessment of whether the synthesized T1 maps preserved disease-specific tissue characteristics in previously unseen subjects.

**Table 1:** Participant demographics and characteristics.

Characteristic	DeepOxyMap dataset	R-GAN dataset
Purpose	Classification & Feature Mapping	T1 Parametric Map Synthesis
Total Subjects (n)	131	2,049
Excluded Subjects (n)	14	23
Final Study Cohort (n)	117 (56 CMR10, 61 CV-SIG)	2,026 (1,871 CV-SIG + 155 CMR10)
Inclusion Criteria	OS-CMR images with confirmed clinical diagnosis (healthy, ICMP, or NICMP) and adequate image quality	Matched OS-CMR and native T1-weighted acquisitions with complete imaging data, including subjects with normal and elevated native T1 values representing fibrosis, infiltration, and different myocardial tissue pathologies
Exclusion Criteria	Image artifacts, incomplete clinical information, or inadequate image quality	Severe imaging artifacts, missing sequences, or slice-matching inconsistencies
Total Images (n)	117 (OS-CMR)	4,052 (pair of OS-CMR and T1- weighted)
Age (years)	53.4 ± 17.2	56.5 ± 10.4
Male, *n* (%)	66 (59.5%)	1,328 (46.5%)
Female, *n* (%)	43 (38.7%)	1,519 (53.2%)

All participants underwent cardiovascular magnetic resonance (CMR) imaging on a 3 T scanner (GE Healthcare) following standardized clinical protocols in accordance with Society for Cardiovascular Magnetic Resonance (SCMR) guidelines, with minor adjustments for breath-hold duration for OS-CMR. For each subject, oxygenation-sensitive CMR (OS-CMR) cine images were acquired during controlled breathing maneuvers, with imaging parameters including a repetition time (TR) of 3.2–4.0 ms, echo time (TE) of 1.5–1.6 ms, flip angle ranging from 50°, and slice thickness of 8 mm, enabling rapid acquisition of myocardial oxygenation dynamics within approximately one minute. In addition, native T1-weighted images were acquired using a modified Look-Locker inversion recovery (MOLLI) sequence with 8 inversion time frames, with inversion times (TIs) ranging from approximately 200–1000 ms to allow reconstruction of T1 recovery curves and quantitative T1 parametric maps. All imaging data were reviewed for quality and consistency before analysis, and corresponding OS-CMR and T1-weighted images were matched on a per-subject basis to ensure accurate pairing for model training and validation.

### 2.2 Preprocessing

All images underwent a standardized preprocessing pipeline to ensure consistency and robustness for model training. Initially, images were resized to 256 × 256 pixels to maintain uniform input dimensions across the dataset. Anatomical correspondence between oxygenation-sensitive CMR (OS-CMR) and their corresponding T1-weighted images was established by identifying and matching equivalent slices for each subject. To further refine spatial alignment while preserving anatomical integrity, rigid image registration (translation and rotation only) was applied, which is particularly suitable for medical imaging as it avoids geometric distortions. Following alignment, contrast enhancement was performed using windowing techniques to improve the visibility of myocardial structures. Image intensities were then normalized to a [0, 1] range, with additional scaling and standardization applied to stabilize training and improve convergence.

Given the limited dataset size, extensive data augmentation was employed to enhance model generalization and robustness. Augmentation strategies included rotations at ±20°, ±40°, ±60°, and ±180°, as well as horizontal and vertical flipping in accordance with medical imaging guidelines. All transformations were applied identically to paired OS-CMR and T1-weighted images to preserve spatial correspondence. The augmented image pairs were subsequently saved and incorporated into the training dataset, effectively increasing data diversity while maintaining anatomical consistency.

#### 2.2.1 ROI selection and clinical assessment

For ROI-based validation, all cases were obtained from clinically labelled datasets in which diagnostic categories and image quality assessments were available from the corresponding clinical reports. Cases affected by severe imaging artifacts or inadequate image quality were excluded prior to analysis. To evaluate the clinical fidelity of the synthesized T1 images, a subset of representative cases from the independent test cohort was selected for detailed ROI-based assessment. Two experienced CMR readers reviewed the available clinical and paraclinical information, including imaging findings and diagnostic reports, to identify myocardial regions with and without evidence of fibrosis or pathological tissue involvement. Using CVI42 software (Circle Cardiovascular Imaging, Calgary, Canada), regions of interest (ROIs) were manually contoured within corresponding myocardial regions on both the original and R-GAN–generated T1 images. The same anatomical locations were used for comparison between reference and synthetic images to ensure consistency.

### 2.3 Training strategy

The proposed framework consists of a two-stage deep learning pipeline, comprising a diagnostic feature extraction model (DeepOxyMap (26)) followed by a novel Residual Generative Adversarial Network (R-GAN) for quantitative image synthesis. In this study, an adapted version of DeepOxyMap was employed using a reduced three-class classification scheme, including ischemic cardiomyopathy (ICMP), non-ischemic cardiomyopathy (NICMP), and healthy controls. The NICMP category encompassed heterogeneous non-ischemic conditions, including hypertrophic cardiomyopathy (HCM) and cardiac amyloidosis.

In the first stage, DeepOxyMap was implemented as a supervised classification model based on a pre-trained VGG19 architecture, fine-tuned for oxygenation-sensitive CMR (OS-CMR) image analysis. The model was trained on a curated dataset of 117 cases. All input images were resized to 256 × 256 pixels and normalized to ensure consistent input representation and stable training. The convolutional base of VGG19, initialized with ImageNet weights, was frozen to leverage transfer learning, while a custom classification head consisting of fully connected layers (512 units, ReLU activation), dropout (0.5), and a final softmax layer was added for multi-class prediction. The dataset was split into training, validation, and test sets, and the model was trained using the Adam optimizer (learning rate = 1 × 10⁻⁴) with categorical cross-entropy loss. To improve generalization and prevent overfitting, early stopping and learning rate reduction on a plateau were applied during training. Model performance was evaluated using accuracy, precision, and recall metrics.

Following the development of DeepOxyMap, a disease-specific validation step was incorporated to assess whether oxygenation-sensitive CMR (OS-CMR) images encode diagnostically relevant myocardial patterns. A subset comprising 10 hypertrophic cardiomyopathy (HCM) and cardiac amyloidosis cases was reserved for independent evaluation. DeepOxyMap was applied to these held-out cases to generate activation-based heatmaps using a dedicated post-processing pipeline developed in this study. This custom pipeline enables the extraction and visualization of discriminative feature regions by projecting deep feature activations onto the original image space, thereby highlighting myocardial areas most relevant for classification decisions.

The generated heatmaps revealed distinct and clinically meaningful spatial patterns within OS-CMR images, including focal regions consistent with fibrosis in HCM and more diffuse myocardial involvement in amyloidosis. These findings suggest that OS-CMR inherently encodes latent tissue characteristics associated with myocardial pathology, which can be uncovered through deep learning–based feature interpretation. Notably, this approach provides a novel mechanism for visualizing diagnostically relevant features directly from contrast-free OS-CMR, offering new insights into its potential for myocardial tissue characterization beyond conventional qualitative assessment. The insights obtained from this intermediate validation step establish a conceptual link between feature extraction and quantitative synthesis, supporting the subsequent use of a Residual Generative Adversarial Network (R-GAN) to generate T1 parametric maps directly from OS-CMR images.

In the second model, the proposed Residual Generative Adversarial Network (R-GAN) is built upon the Pix2Pix framework, with architectural modifications designed to enhance feature learning capacity, improve training stability, and mitigate vanishing gradient effects. Specifically, residual blocks were incorporated into both the generator and discriminator networks, with the optimal configuration determined through hyperparameter tuning, resulting in nine residual blocks in the generator and three in the discriminator. The generator adopts an encoder–decoder architecture that enables progressive feature abstraction and reconstruction, while residual connections facilitate efficient gradient propagation and stable convergence. The discriminator follows a PatchGAN architecture, allowing the model to evaluate image realism at the patch level, thereby capturing both global anatomical structures and fine-grained local textures critical for accurate medical image synthesis. To optimize training and ensure both perceptual and structural fidelity of the generated T1-weighted images, a composite loss function was employed. The adversarial loss drives the generator to produce outputs indistinguishable from real T1 images, as assessed by the discriminator. To further enhance high-level feature consistency, a perceptual loss (ℒ_VGG) based on feature maps extracted from a pre-trained VGG19 network was incorporated, enabling comparison in a deep feature space rather than solely at the pixel level. Additionally, an L1 reconstruction loss (ℒ_L1) was applied to enforce pixel-wise similarity between generated and ground-truth images, preserving structural alignment and reducing blurring artifacts. The model was trained using the Adam optimizer with a learning rate of 2 × 10⁻⁴ and a momentum parameter (*β*₁) of 0.5. Training was conducted for approximately 150,000 iterations, with model checkpoints saved every 5,000 steps and training progress monitored using TensorBoard for loss convergence and performance tracking. The training process was completed in approximately two hours on an NVIDIA A100 GPU, while inference during testing required approximately 40 s per case.

### 2.4 Evaluation metrics

The proposed two-stage framework was evaluated using a combination of classification metrics, image similarity measures, and clinically grounded validation techniques to assess both diagnostic relevance and quantitative fidelity. For the DeepOxyMap model, classification performance was assessed using accuracy, precision, recall, and area under the receiver operating characteristic curve (AUC–ROC). In addition, activation-based heatmaps were extracted to verify that the model focused on anatomically relevant myocardial regions rather than non-specific image features, thereby providing qualitative evidence against overfitting and confirming that learned representations correspond to clinically meaningful areas associated with disease patterns.

For the generative stage, the performance of the Residual GAN (R-GAN) was quantitatively compared with the Pix2Pix baseline using standard image similarity metrics, including Structural Similarity Index Measure (SSIM), Peak Signal-to-Noise Ratio (PSNR), and Pearson Correlation Coefficient (PCC), which respectively assess structural fidelity, noise characteristics, and linear agreement between synthetic and ground-truth T1-weighted images.

To ensure clinical validity, a multi-level evaluation pipeline was implemented. First, signal intensity (SI) patterns were analyzed by CVI42, allowing direct comparison between synthetic and original images. Second, a physics-informed validation was performed using Extended Phase Graph (EPG)–based simulations using an MRI dictionary, where the synthetic images were compared against theoretical T1 recovery models derived from MRI signal equations. Third, region-of-interest (ROI)-based T1 recovery curves were extracted from both real and synthetic across different myocardial regions, and their agreement was evaluated both visually and quantitatively.

Quantitative agreement between synthetic and original T1 curves was assessed using Mean Absolute Error (MAE), Mean Squared Error (MSE), Root Mean Squared Error (RMSE), coefficient of determination (*R*^2^), and both Pearson and Spearman correlation coefficients. Finally, colour-coded T1 parametric maps were generated using CVI42 for qualitative assessment of spatial patterns, enabling direct visualization of myocardial tissue characteristics and comparison of pathological regions between synthetic and reference images.

## 3 Experimental results

**Figure 1:**
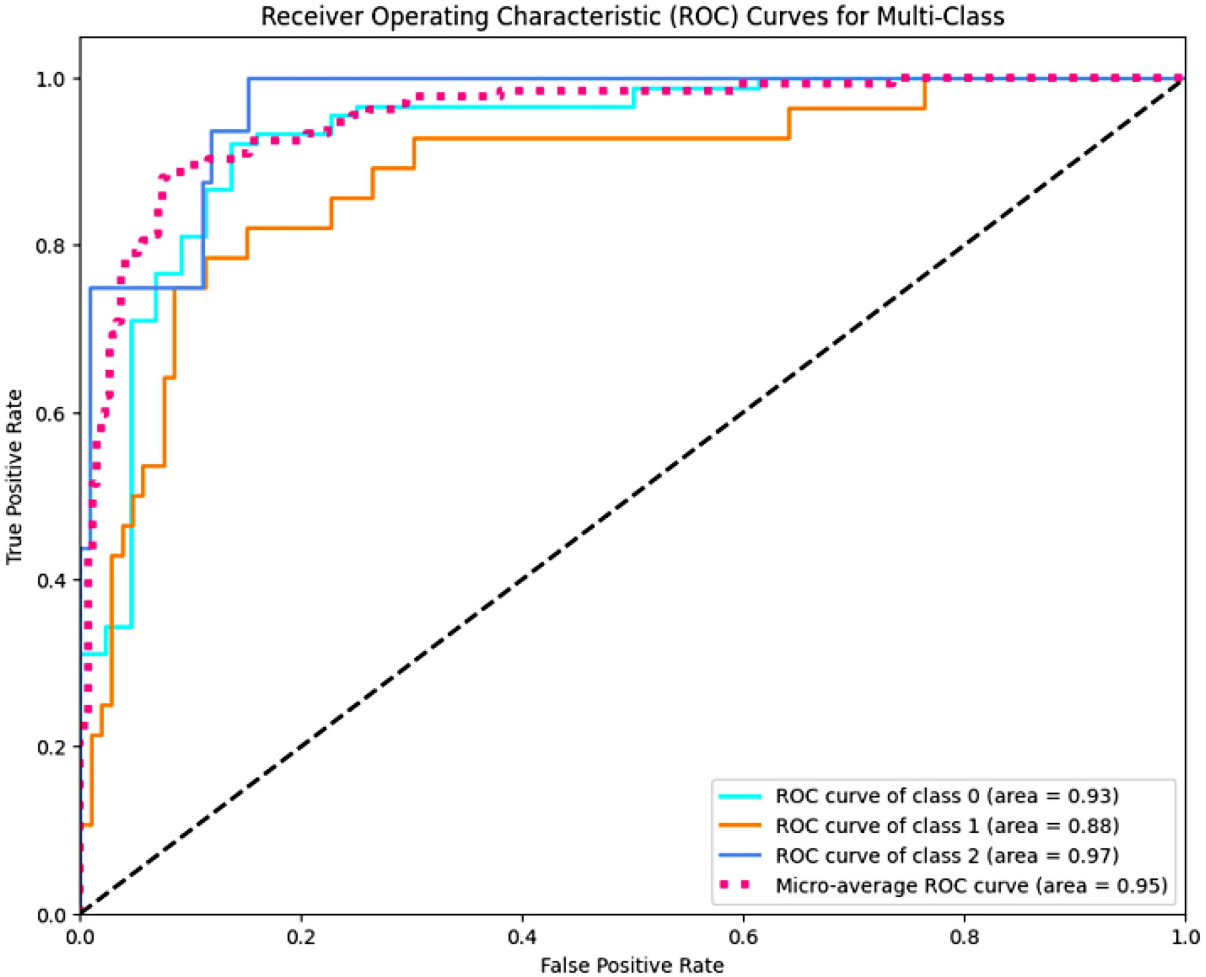
ROC curves for DeepOxyMap classification. High AUC values (Micro-average: 0.95) demonstrate strong discrimination between ICMP, NICMP, and healthy classes.

The DeepOxyMap model demonstrated strong classification performance in distinguishing between different cardiomyopathies. The model achieved an accuracy of 83.5%**,** with a precision of 85.5% and a recall of 79.7%**.** Receiver operating characteristic (ROC) analysis further confirmed robust discriminative capability, with area under the curve (AUC) value of 0.95 average**,** 0.93 for ICMP**,** 0.88 for NICMP**,** 0.97 for healthy controls (Figure 1)**.**

**Figure 2:**
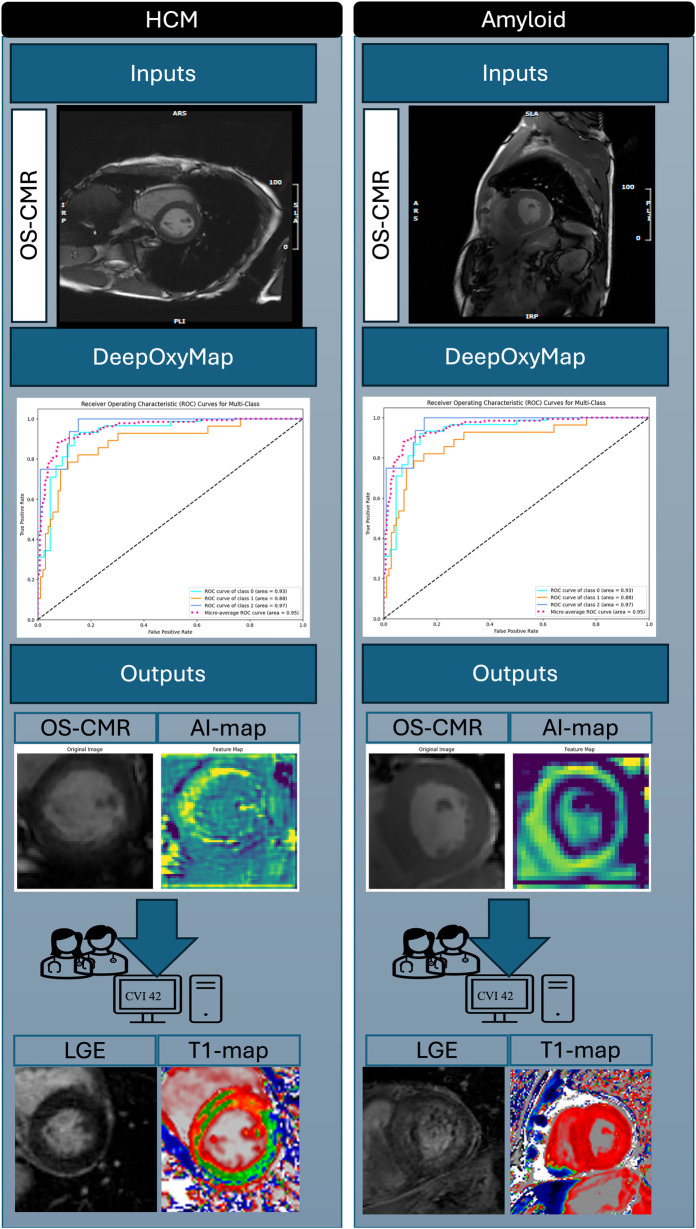
Deepoxymap framework and feature interpretation using activation heatmaps. Oxygenation-sensitive CMR (OS-CMR) images are used as input to the DeepOxyMap model for multi-class classification of myocardial conditions. The model demonstrates high discriminative performance, as illustrated by the receiver operating characteristic (ROC) curves (AUC ≈ 0.95). Activation-based heatmaps (AI-maps) are generated to visualize regions contributing to the model's decision, highlighting diagnostically relevant myocardial areas. Representative examples show that the model consistently focuses on anatomically meaningful regions within the myocardium, supporting its ability to capture disease-specific features from contrast-free OS-CMR images.

Beyond classification metrics, activation-based heatmaps were generated to interpret the model's decision-making process. These heatmaps consistently localized on anatomically relevant myocardial regions rather than background structures, supporting that the model did not rely on spurious correlations. In representative cases, focal activation patterns were observed in hypertrophic cardiomyopathy (HCM), while diffuse myocardial involvement was identified in cardiac amyloidosis. This provides qualitative evidence that OS-CMR encodes disease-specific myocardial features that can be effectively extracted using deep learning (Figure 2).

Following the identification of disease-specific myocardial patterns using DeepOxyMap, the second stage of the framework was developed, in which a Residual Generative Adversarial Network (R-GAN) was employed to synthesize T1-weighted maps directly from OS-CMR images.

Table 2 presents a quantitative comparison of the synthesized T1-weighted images generated by different models, including Pix2Pix and the proposed R-GAN, evaluated using standard image similarity metrics. These metrics provide an objective assessment of structural fidelity, signal consistency, and overall image quality.

**Table 2:** Quantitative comparison of synthesized T1 maps across different models using image similarity metrics. R-GAN with augmentation significantly outperformed other methods in terms of SSIM (structural similarity index), PSNR (peak signal-to-noise ratio), and PCC (Pearson correlation coefficient), indicating superior visual and signal fidelity.

Metric	RGAN with augmentation	RGAN without augmentation	Pix2Pix with augmentation	Pix2Pix without augmentation
Average SSIM	0.805	0.766	0.254	0.158
Average PSNR	16.1	15.21	15.03	8.84
Average PCC	0.748	0.678	0.231	0.03

The evaluation metrics include Structural Similarity Index Measure (SSIM), Peak Signal-to-Noise Ratio (PSNR), and Pearson Correlation Coefficient (PCC), which collectively assess perceptual image quality, signal fidelity, and linear correspondence with ground truth images.

Among all evaluated models, the R-GAN trained with data augmentation demonstrated superior performance. Specifically, it achieved an SSIM of 0.805, a PSNR of 16.1 dB, and a PCC of 0.748, indicating a high degree of structural similarity, reduced noise, and strong linear correlation with the reference T1 maps. In contrast, the baseline Pix2Pix model yielded significantly lower values, with an SSIM of 0.254, PSNR of 15.03 dB, and PCC of 0.231, highlighting its limited capacity to preserve both anatomical and signal fidelity in the generated outputs (Figure 1). These findings confirm that the inclusion of residual blocks, perceptual loss, and robust augmentation strategies contributes substantially to improved synthetic image quality in the R-GAN framework.

To validate the tissue-level fidelity of the synthetic images, we imported the R-GAN-generated T1-weighted images into CVI software and performed quantitative signal intensity (SI) analysis on regions of interest (ROIs). Figure 2 illustrate the results from two representative cases: amyloid, and non-ischemic cardiomyopathies. For each case, signal intensities were measured in two ROI myocardial regions from the original and synthesized images. In the amyloid case, the fibrotic region in ROI-1 measured 1476 (original) and 1371 (synthesized), while ROI-2 showed 1356 (original) and 1252 (synthesized). In the non-ischemic case, both ROIs were fibrotic, showing 951 and 825 in the original image, and 904 and 904 in the synthesized counterpart.

To validate the physiological consistency of the synthesized T1 signals, an Extended Phase Graph (EPG) using MRI dictionary-based simulation was performed and compared against both original and R-GAN–generated T1 recovery curves. As summarized in Table 3, the synthetic signals demonstrated near-perfect agreement with theoretical and reference curves across both hypertrophic cardiomyopathy (HCM) and cardiac amyloidosis cases. For non-ischemic (HCM) cases, the model achieved a Mean Absolute Error (MAE) of 0.0069, Root Mean Squared Error (RMSE) of 0.0125, and a coefficient of determination (*R*^2^) of 0.9958, with Pearson and Spearman correlation coefficients of 0.998 and 1.000, respectively. Similarly, for amyloidosis, the agreement was even stronger, with *R*^2^ = 0.9994 and perfect correlation values (Pearson = 1.000, Spearman = 1.000), indicating an almost exact reconstruction of the underlying T1 recovery behaviour.

**Table 3:** EPG-based simulation validation of T1 recovery curves.

Pathology type	MAE	MSE	RMSE	*R* ^2^	Pearson	Spearman
Non-Ischemic (HCM)	0.0069	0.0002	0.0125	0.9958	0.998	1.000
Amyloid	0.00001	0.0049	0.0025	0.9994	1.000	1.000

**Figure 3:**
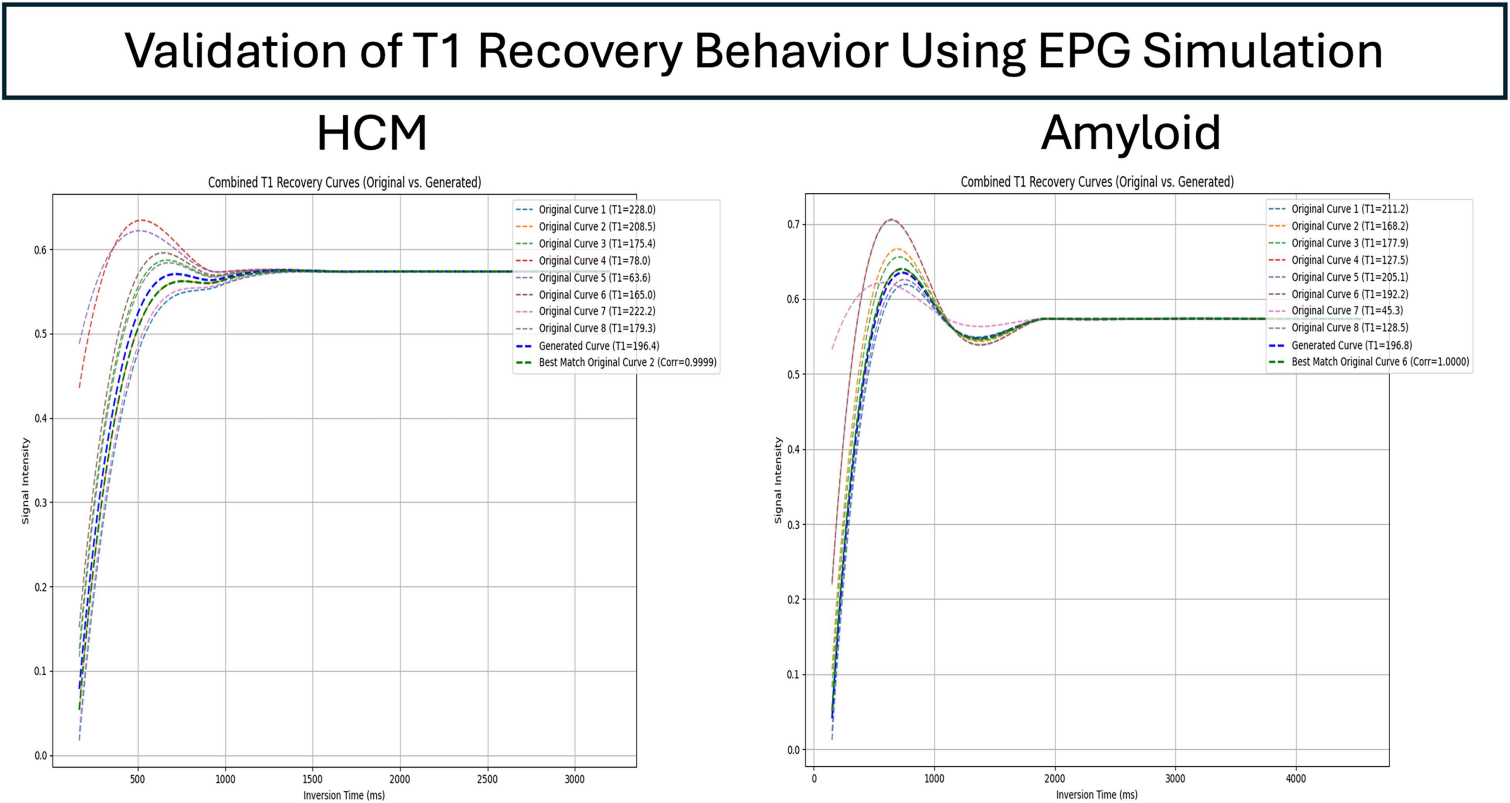
EPG-based comparison of T1 recovery curves between synthetic and reference images in HCM and amyloidosis cases.

Figure 3 further illustrates the comparison of T1 recovery curves across representative amyloidosis cases, demonstrating that the generated curves closely follow both the original measurements and the EPG-derived theoretical models. This strong agreement confirms that the proposed R-GAN does not merely reproduce visual features but accurately captures the underlying MRI signal physics.

Importantly, the preservation of distinct recovery curve characteristics between HCM and amyloidosis supports the model's ability to maintain disease-specific signal signatures. While HCM cases exhibited characteristic recovery behaviour associated with localized myocardial alterations, amyloidosis demonstrated patterns consistent with diffuse tissue involvement. This distinction highlights the potential of the proposed framework to assist in clinical differentiation between cardiomyopathies using contrast-free OS-CMR–derived synthetic T1 maps.

To further evaluate the clinical reliability of the synthesized T1-weighted images, a region-of-interest (ROI)–based analysis was performed using CVI42 software. Quantitative comparison between synthetic and reference T1 recovery curves demonstrated strong agreement across both hypertrophic cardiomyopathy (HCM) and cardiac amyloidosis cases, as summarized in Table 4.

**Table 4:** ROI-based evaluation of T1 recovery curves (CVI analysis).

Pathology type	MAE	MSE	RMSE	*R* ^2^	Pearson	Spearman
Amyloid	0.0313	0.0079	0.0886	0.9139	0.9619	0.8571
Non-Ischemic (HCM)	0.0294	0.0023	0.0483	0.9720	0.9863	0.9286

For amyloidosis, the model achieved a Mean Absolute Error (MAE) of 0.0313, Root Mean Squared Error (RMSE) of 0.0886, and a coefficient of determination (*R*^2^) of 0.9139, with a Pearson correlation of 0.9619 and Spearman correlation of 0.8571. For non-ischemic (HCM) cases, performance was further improved, with MAE = 0.0294**,** RMSE = 0.0483, and *R*^2^ = 0.9720, alongside strong correlation values (Pearson = 0.9863**,** Spearman = 0.9286), indicating a high level of agreement between synthetic and original T1 signal evolution.

**Figure 4:**
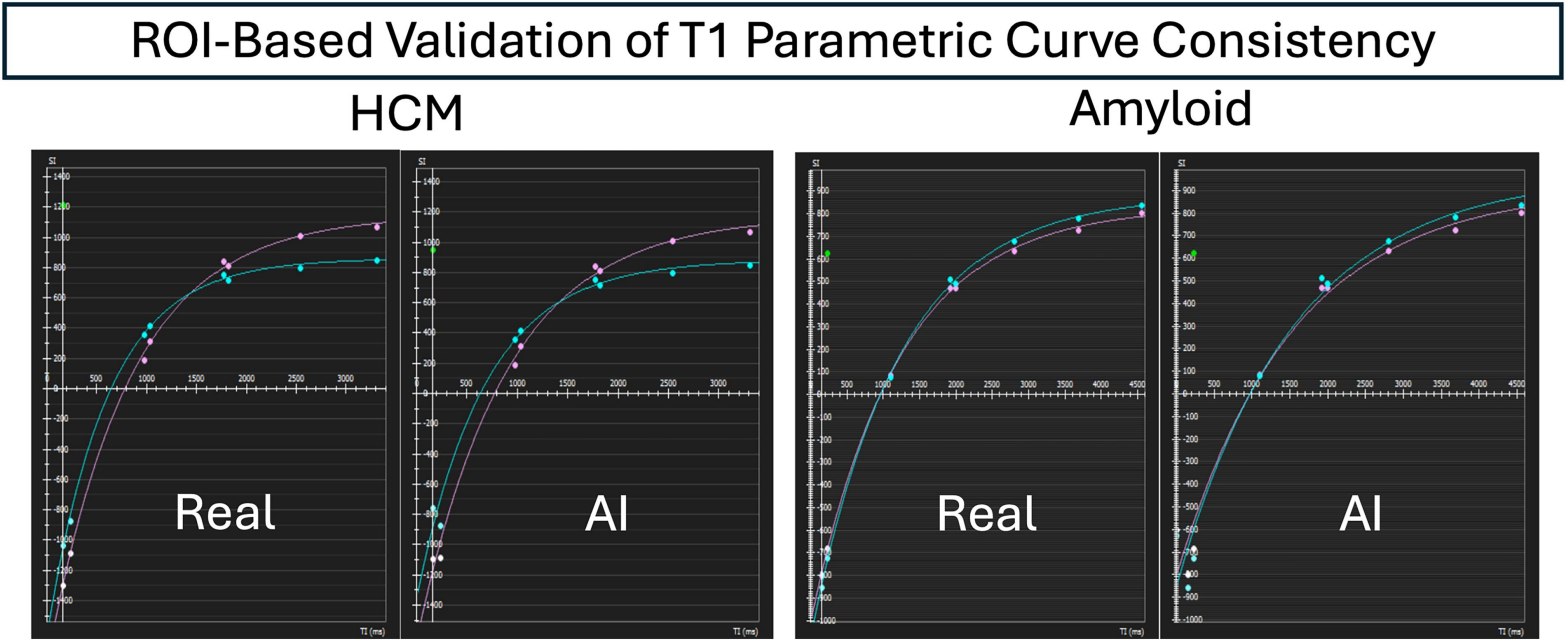
ROI-based comparison of T1 recovery curves between original and R-GAN–generated images.

Representative examples of ROI-based T1 recovery curves are shown in Figure 4, where synthetic curves closely follow the trajectory of the corresponding reference curves across inversion times. Minor deviations observed in amyloidosis cases likely reflect the increased heterogeneity of diffuse myocardial involvement, yet overall curve morphology and recovery behaviour remain well preserved.

Importantly, this ROI-based validation complements the EPG-based physics analysis by demonstrating that the generated signals are not only theoretically consistent but also clinically meaningful when evaluated in patient-specific myocardial regions. The ability of the model to preserve disease-specific signal patterns within anatomically defined ROIs further supports its potential utility for contrast-free myocardial tissue characterization and differentiation between cardiomyopathies.

**Figure 5:**
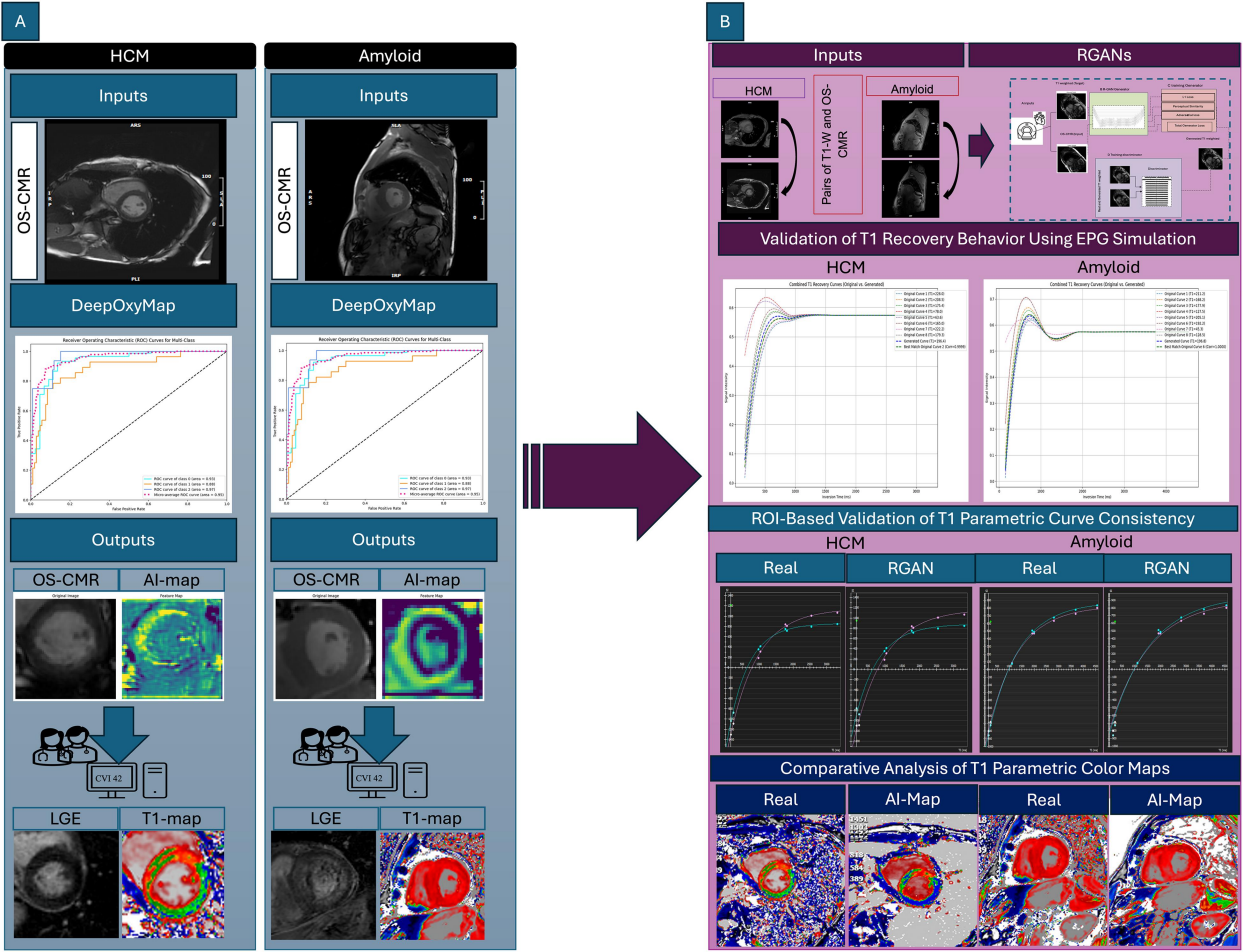
**Overview of the proposed DeepOxyMap–R-GAN framework. (A)** modify DeepOxyMap was used to extract fibrosis-related features from oxygenation-sensitive CMR (OS-CMR) images and generate AI-derived tissue maps, demonstrated in representative hypertrophic cardiomyopathy (HCM) and cardiac amyloidosis cases. **(B)** R-GAN framework for synthetic T1 map generation from OS-CMR images with multi-level clinical validation.

To further assess the clinical interpretability of the synthesized outputs, colour-coded T1 parametric maps were generated from both original and R-GAN–derived images using CVI42 software. Visual comparison demonstrated strong agreement between synthetic and reference maps across all evaluated cases. In cardiac amyloidosis, the synthetic maps consistently reproduced diffuse myocardial involvement, characterized by globally elevated T1 values represented by widespread red regions across the myocardium. In contrast, hypertrophic cardiomyopathy (HCM) cases exhibited more localized areas of elevated T1 values corresponding to focal fibrosis, while surrounding myocardial regions remained relatively preserved. Healthy myocardial tissue, when present, demonstrated lower T1 values and appeared predominantly in green regions (Figure 5).

**Figure 6:**
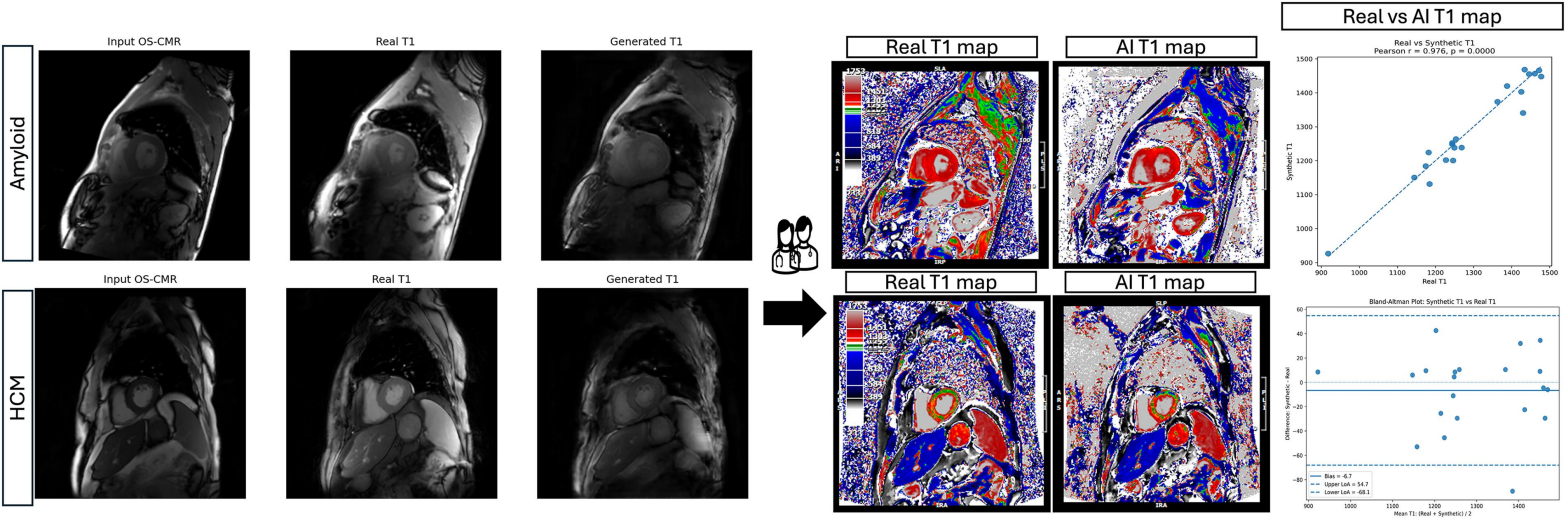
Qualitative and quantitative validation of synthetic T1 maps generated from OS-CMR in cardiomyopathy patients. Representative examples from patients with cardiac amyloidosis (top row) and hypertrophic cardiomyopathy (bottom row) are shown. Left panels demonstrate the input OS-CMR image, corresponding native T1 map, and AI-generated synthetic T1 image. Middle panels show quantitative T1 maps generated from the native and synthetic images and analyzed independently using CVI42 with identical contours and regions of interest (ROIs). Segmental T1 values were extracted from matching myocardial ROIs in both native and synthetic T1 maps for quantitative comparison. Right panels summarize agreement across the validation cohort using correlation and Bland–Altman analyses. Synthetic T1 measurements demonstrated a strong correlation with native T1 values (Pearson *r* = 0.976, *p* < 0.001), with a mean bias of −6.7 ms and 95% limits of agreement from −68.1 to 54.7 ms, indicating good quantitative agreement and preservation of clinically relevant myocardial tissue characteristics.

In the independent HCM and cardiac amyloidosis validation cohort (*n* = 21), Bland–Altman analysis demonstrated excellent agreement between native and synthetic T1 values, with a mean bias of −6.69 ms and 95% limits of agreement ranging from −68.11 to 54.72 ms (Figure 6). The 95% confidence interval of the bias was −20.95–7.57 ms, indicating minimal systematic measurement error. Native and synthetic T1 values were highly correlated (Pearson r = 0.976, *p* < 0.001), further supporting the quantitative fidelity of the generated maps.

## 4 Discussion

This study presents a unified, contrast-free framework for myocardial tissue characterization by combining DeepOxyMap-based feature extraction with R-GAN–based quantitative T1 map synthesis. To our knowledge, this is the first study to demonstrate, through deep learning–based feature interpretation, that fibrosis-related information can be directly visualized within oxygenation-sensitive CMR (OS-CMR) without the use of contrast agents.

A key finding of this work is that OS-CMR inherently encodes diagnostically relevant myocardial information. As demonstrated in the Results, DeepOxyMap achieved strong classification performance (accuracy up to 83% and AUC up to 0.95) and generated activation-based heatmaps that consistently localized to the myocardium (Figures 1, 5). These results indicate that OS-CMR contains latent fibrosis-related features that can be extracted directly, supporting its potential as a contrast-free modality for myocardial tissue characterization.

This observation addresses an important and previously unresolved question in cardiac imaging: whether non-contrast cine sequences can reflect tissue-level pathology typically assessed by LGE or T1 mapping. The heatmap analysis (Figure 1) demonstrates that DeepOxyMap identifies disease-specific spatial patterns, including focal myocardial involvement in hypertrophic cardiomyopathy and diffuse patterns in cardiac amyloidosis, supporting the hypothesis that OS-CMR encodes clinically meaningful tissue characteristics.

Building on this insight, the second stage of the framework employs a Residual GAN (R-GAN) to synthesize quantitative T1 maps directly from OS-CMR. As demonstrated in Table 2, the proposed R-GAN significantly outperforms the Pix2Pix baseline, achieving higher structural similarity (SSIM = 0.805), improved signal fidelity (PCC = 0.748), and better noise characteristics (PSNR = 16.1 dB). These results indicate that the model effectively preserves both anatomical structure and signal relationships between OS-CMR and T1-weighted images.

Importantly, the clinical validity of the synthesized maps is supported by multi-level validation. First, EPG-based simulation results (Table 3, Figure 3) show near-perfect agreement between synthetic and theoretical T1 recovery curves (*R*^2^ up to 0.9994, Pearson up to 1.000), confirming that the generated signals follow underlying MRI physics rather than merely reproducing visual features. Second, ROI-based analysis using CVI42 (Table 4 and Figure 4) demonstrates strong agreement between synthetic and original T1 curves (Pearson > 0.96), indicating that the model preserves clinically relevant signal behavior within anatomically defined myocardial regions.

Finally, visual comparison of T1 parametric color maps (Figure 6) further confirms the model's ability to maintain disease-specific tissue patterns. Synthetic maps accurately reproduce diffuse myocardial involvement in amyloidosis and focal fibrosis patterns in HCM, consistent with known pathological characteristics. This is particularly relevant for amyloidosis, where conventional LGE imaging often struggles due to diffuse enhancement patterns. The ability of the proposed framework to capture these differences suggests its potential utility for improving non-contrast diagnosis of cardiomyopathies.

Compared to existing approaches such as Virtual Native Enhancement (VNE) (18), which require multi-sequence inputs including native T1 maps, our method operates using a single OS-CMR acquisition. This significantly simplifies the imaging workflow while providing full pixel-wise quantitative maps rather than segment-based predictions. Direct comparison with previously reported synthetic T1 mapping approaches remains challenging, as no prior clinical CMR study has investigated the direct generation of quantitative T1 maps from oxygenation-sensitive CMR images. Existing AI-based cardiac tissue characterization frameworks, such as Virtual Native Enhancement (VNE), rely on the integration of multiple imaging modalities, including cine CMR, native T1 mapping, and LGE imaging. In contrast, the proposed framework utilizes oxygenation-sensitive CMR as the sole input modality, aiming to extract and translate latent tissue information into quantitative T1 maps without requiring additional sequences. Pix2Pix was selected as the primary benchmark because the proposed framework addresses a paired image-to-image translation problem, where each OS-CMR image is directly mapped to a corresponding T1-weighted image. While alternative generative architectures, including conditional GANs, super-resolution networks, and diffusion models, have demonstrated promising performance in other medical imaging applications, many of these approaches were developed for different objectives such as data augmentation, image enhancement, or unconditional image generation. Furthermore, recent studies have highlighted the potential for diffusion models to exhibit memorization of training data (27), particularly in medical imaging settings involving limited or highly homogeneous datasets, raising concerns regarding both generalizability and privacy preservation. Therefore, for this proof-of-concept study, we selected Pix2Pix as the most established and clinically relevant benchmark for paired image-to-image synthesis and evaluated whether residual learning could further improve performance within this framework.

From a methodological perspective, the improved performance of R-GAN can be attributed to the use of residual connections, perceptual loss, and PatchGAN discrimination, which together enable accurate reconstruction of both global and local image features. While diffusion-based models have shown promise in image synthesis, their computational complexity and slow inference limit their current applicability in cardiac MRI. In contrast, the proposed R-GAN framework is fast, deterministic, and more suitable for clinical deployment.

## 5 Limitations and future work

Despite the promising results, several limitations should be acknowledged. First, the model was developed using data acquired at a single center and on a single scanner platform. Although two independent cohorts were included, additional validation across multiple institutions, scanner vendors, acquisition protocols, and broader patient populations is necessary to establish the generalizability and robustness of the proposed framework. Future studies will therefore focus on multi-center validation encompassing greater demographic and pathological variability.

Second, the current implementation is based on single-slice analysis, which does not fully capture the three-dimensional structure of the myocardium. Extending the framework to whole-heart, multi-slice, and multi-phase acquisitions will be essential for comprehensive clinical integration.

Third, although the model demonstrates strong agreement with ground-truth T1 maps, minor discrepancies were observed in regions affected by motion or imaging artifacts. Further investigation is required to understand the physiological implications of these differences.

Fourth, the model was trained using data from a single scanner and acquisition protocol. Validation across different vendors, field strengths, and imaging settings is necessary to ensure robustness and clinical applicability.

Although the disease-specific validation cohorts for hypertrophic cardiomyopathy (HCM) and cardiac amyloidosis were relatively limited in size, several measures were implemented to mitigate the impact of this limitation. DeepOxyMap employed transfer learning from a pre-trained VGG19 architecture together with data augmentation strategies to improve feature learning and reduce overfitting in the presence of limited training data. In addition, activation-based heatmaps were used to verify that the model focused on anatomically relevant myocardial regions rather than non-specific image features, providing further evidence that the learned representations reflected clinically meaningful tissue characteristics. Nevertheless, the disease-specific validation results should be considered preliminary. Larger cohorts and multi-center studies will be required to further validate the robustness, generalizability, and clinical applicability of the proposed framework across diverse cardiomyopathy populations.

Future work will also explore disease-specific model training, integration with segmentation and classification networks, and extension to additional modalities such as T2 mapping or oxygenation-sensitive imaging synthesis. The incorporation of super-resolution techniques may further enhance spatial detail and diagnostic utility.

## 6 Conclusion

Our results indicate that oxygenation-sensitive CMR (OS-CMR) inherently encodes latent information related to myocardial fibrosis and tissue composition, which can be effectively extracted and visualized using deep learning algorithms. These features can be translated into high-fidelity T1 maps, suggesting the potential for contrast-free myocardial tissue characterization and providing a foundation for future investigation of alternative imaging strategies. The generated maps preserve both structural and disease-specific signal patterns, enabling differentiation between hypertrophic cardiomyopathy and cardiac amyloidosis.

By integrating feature discovery, quantitative synthesis, and multi-level validation within a single framework, this approach provides a practical pathway toward simplified, contrast-free cardiac MRI workflows. While the results are encouraging, the proposed framework remains investigational and requires validation in larger prospective and multi-center studies before clinical implementation. Nevertheless, the findings support the potential of artificial intelligence–based contrast-free myocardial tissue characterization as a future complementary tool in cardiovascular magnetic resonance imaging.

By integrating feature discovery, quantitative synthesis, and multi-level validation within a single framework, this approach provides a practical pathway toward simplified, contrast-free cardiac MRI workflows. Although further prospective and multi-center validation will be important to establish broader generalizability, the encouraging results presented in this study demonstrate the potential of combining artificial intelligence with oxygenation-sensitive CMR for contrast-free myocardial tissue characterization. These findings support the growing role of AI in non-invasive myocardial tissue assessment and may contribute to the future development of more accessible and efficient cardiovascular magnetic resonance workflows.

## Data Availability

The original contributions presented in the study are included in the article/Supplementary Material, further inquiries can be directed to the corresponding authors.
